# False negative antigen tests in dogs infected with heartworm and placed on macrocyclic lactone preventives

**DOI:** 10.1186/s13071-015-0698-4

**Published:** 2015-02-04

**Authors:** Jason Drake, Jeff Gruntmeir, Hannah Merritt, Lynn Allen, Susan E Little

**Affiliations:** Novartis Animal Health, Greensboro, NC USA; Department of Veterinary Pathobiology, Center for Veterinary Health Sciences, Oklahoma State University, Room 250 74078 McElroy Hall, Stillwater, OK USA; Swaim Serum Company, Oklahoma City, OK USA

**Keywords:** *Dirofilaria immitis*, False-negative antigen test, Immune complexes, Slow kill

## Abstract

**Background:**

Dogs with chronic inflammation, including those with heartworm being managed with macrocyclic lactones and doxycycline (slow kill, SK), may develop immune complexes that block detection of *Dirofilaria immitis* antigen on commercial tests.

**Methods:**

To determine if SK could result in development of false-negative antigen tests, we collected serum samples from dogs that had been diagnosed with heartworm by antigen detection, with or without confirmation by detection of *D. immitis* microfilariae, placed on monthly macrocyclic lactones and doxycycline, and that later tested negative on an antigen test, and then tested them for antigen of *D. immitis* before and after treatment to disrupt immune complexes.

**Results:**

Serum samples from a total of 15 dogs managed with SK were negative for antigen prior to heating on commercial assay (DiroCHEK®, Zoetis) by colorimetric detection and spectrophotometry, but after heat treatment, 8/15 (53.3%) samples converted to positive. Review of the medical records of each dog indicated that, after the heartworm diagnosis, only 7/15 (46.7%) dogs appeared to receive preventive monthly as prescribed, including 3 dogs that had detectable antigen after heating the sample and 4 dogs that did not have detectable antigen after heating. Whole blood was available from 9 dogs; microfilariae of *D. immitis* were detected in 1 sample.

**Conclusions:**

These data suggest that immune complex formation in dogs infected with heartworm and managed with SK can induce false negative antigen test results, misleading veterinarians and owners about the efficacy of this approach. Moreover, compliance with preventive administration appears poor, even after a heartworm diagnosis. The presence of persistent microfilaremia in at least one dog has implications for resistance selection.

## Background

Slow kill (SK) is defined as the use of monthly macrocyclic lactone heartworm preventives, with or without doxycycline, in lieu of melarsomine dihydrochloride (Immiticide®, Merial) in dogs infected with adult *Dirofilaria immitis* [1]. Historically, veterinarians have used SK to manage adult heartworm infections for a variety of reasons, including when the approved adulticide is not available, when the cost of adulticide treatment is rejected by a dog owner, or when the medical risk of adulticide therapy is thought to be too great. The practice of SK is explicitly discouraged within the guidelines of both the American Heartworm Society (AHS) and the Companion Animal Parasite Council (CAPC) [1,2]. The European Scientific Counsel Companion Animal Parasites (ESCCAP) also explicitly does not recommend the use of SK-like protocols [3].

Several reasons to avoid SK have been described, including compliance failure and selection for resistance [4]. The adult worms, which may be > 30 cm in length, reside in the pulmonary arteries and cause severe inflammation, truncation of pulmonary vessels, thickening of the arterial parenchyma, pulmonary hypertension and right heart failure [5-9]. Utilization of the approved adulticide, melarsomine dihydrochloride, is an effective method for elimination of adult heartworms [10], while SK methods of continuous monthly use of preventive doses of macrocyclic lactones, with or without the addition of doxycycline, result in worm death and elimination over a lengthy and somewhat unpredictable period of time [11,12]. Efficacy of SK is poor (56.3%) against 8-month-old worms when monthly low-dose ivermectin is administered for 16 consecutive months [11] but improves (94.9%) when administered consistently for 29 consecutive months [12]. However, SK allows worms to continue to reside within the pulmonary arteries, prolonging the inflammatory process and allowing further disease progression [4]. Indeed, heartworm-infected dogs administered SK for a one-year period developed radiographic signs of heartworm disease and severe pulmonary pathology with multiple pathologic changes including enlarged pulmonary arteries, villous proliferation characteristic of heartworm infection, alveolar disease, interstitial lung disease, and parenchymal fibrosis [4].

In clinical practice and in clinical research projects, veterinarians may use antigen testing as a proxy for SK efficacy [13,14]. The relationship between antigen status, microfilaremia, and the presence of adult worms in dogs managed with SK has not been well defined. Interestingly, in the original publication describing the prolonged death of adult heartworms during monthly administration of preventive, antigen levels decreased during administration of ivermectin to heartworm-infected dogs even though worms survived, suggesting the process of SK interferes with either antigen production by *D. immitis* or antigen detection by commercial tests [11].

All currently available heartworm preventives contain macrocyclic lactones as the active ingredient against *D. immitis* [15]. Recently, *D. immitis* resistant to macrocyclic lactone preventives have been described in North America [16-18]. Repeated administration of preventives to dogs infected with adult *D. immitis* may increase the risk of resistance to macrocyclic lactones by increasing selective pressure, placing all dogs, including uninfected dogs maintained on preventives, at higher risk of infection [15]. Although experimental data document that SK methods require more than 1–2 years to eliminate adult heartworms [11,12], some veterinarians have reported dogs converting from *D. immitis* antigen positive to antigen negative status within a few months of starting SK (S. Little, pers. comm.). Recent publications document false negative antigen tests occur in both dogs and cats, presumably due to formation of immune complexes, a phenomenon described in the literature through the 1980s [19,20]. Here we report initial data indicating false negative antigen tests are common in dogs on SK.

## Methods

### Source of samples

Serum and EDTA-anticoagulated whole blood samples were requested from canine patients seen by general practitioners that had been previously diagnosed by the participating veterinary practice as infected with *D. immitis* by a commercial in-clinic antigen test, with or without confirmation by microfilaria testing, within the previous 36 months. Specifically, samples were requested from dogs that had not been treated with the label-approved adulticide (melarsomine dihydrochloride), but instead were placed on a monthly macrocyclic lactone-based preventive and doxycycline (SK). A history was collected for each dog and included patient identification information, previous heartworm testing method(s), test timing, test result(s), SK protocol employed, heartworm preventive used, and compliance with heartworm prevention. Compliance with heartworm prevention was evaluated based on date of purchase of heartworm preventive from the clinic. The research protocol was approved by the Novartis Animal Welfare Officer prior to instituting the research and conformed to all requirements and ethical guidelines of both Novartis Animal Health and Oklahoma State University’s Center for Veterinary Health Sciences.

### Inclusion and exclusion criteria

Samples included in this study were collected from dogs with a diagnosis of heartworm infection in the past 36 months based on commercial antigen test and that had converted to antigen-negative status within the past 24 months using any commercially approved in-clinic antigen test conducted following the manufacturer’s instructions. Serum samples were tested upon receipt to confirm antigen-negative status (see below); only samples confirmed negative upon receipt were included in the study. Samples from dogs treated with melarsomine dihydrochloride and those that could not be confirmed through the medical records to meet the inclusion requirements were excluded from the study. Serum samples (3–7 mL) were required from each dog enrolled in the study. When available, EDTA-anticoagulated whole blood was also evaluated for microfilariae (see below) but submission of a paired whole blood sample was not required for inclusion.

### Antigen testing

Upon receipt of each serum sample, antigen negative status was confirmed on a microtiter well based assay (DiroCHEK®, Zoetis) according to manufacturer’s instructions. Only samples that tested negative upon initial evaluation were included in the study. Following confirmation testing, each sample was heat treated and retested as previously described [21]. Briefly, 1 mL of serum was placed in a 1.5 mL snap-cap microcentrifuge tube, heated to 104°C for 10 minutes in a standard laboratory dry heat block, and the resulting coagulum centrifuged at 16,000 × g for 5 minutes. After centrifugation, the supernatant was removed from the coagulum and tested in triplicate using a microtiter well based assay (DiroCHEK®, Zoetis) according to manufacturer’s instructions. In addition to colorimetric change, spectrophotometry (650 nm) was used to record the optical density (O.D.) of each individual sample, before and after heat treatment, on the same microtiter plate using the same positive and negative controls and identical calculated cut-off values [21].

### Microfilaria testing

Modified Knott tests were performed on all dogs for which whole blood samples were available as previously described [22]. Briefly, 9 mL of 2% formalin was added to 1 mL of whole blood, the cells allowed to lyse, the mixture centrifuged at 1,200 × g, and the pellet stained with methylene blue and examined by microscopy for microfilariae. Identify of microfilariae were confirmed by morphology and by PCR and sequencing as previously described [22,23].

## Results

A total of 15 dogs met the enrollment inclusion and exclusion criteria. Serum samples were submitted from all 15 dogs. Antigen testing of the samples post-heating revealed serum from 8 of 15 dogs (53.3%) that had become antigen negative following SK therapy were actually still antigen positive; samples from 7 of 15 (46.7%) dogs remained antigen negative after heating (Table 1). Paired anticoagulated whole blood samples were submitted from 9 of 15 dogs; of these, 1/9 (11.1%) had evidence of microfilariae. Microfilariae were confirmed as *D. immitis* via morphology and PCR followed by sequencing (data not shown).

**Table 1:** **Prescribed preventive, administration route, compliance, doxycycline use, and results of modified Knott test and antigen tests on samples collected from 15 heartworm infected dogs managed with slow kill**

**Dog ID**	**Preventive prescribed**	**Route of administration**	**Compliant with dispensing**	**Doxycycline (10 mg/kg PO) protocol**	**Modified Knott**	**Pre-heating antigen test results**	**Post-heating antigen test results**
1	Moxidectin	Topical	No	30d	ND	-	+
2	Moxidectin	Topical	Yes	30d, four times	ND	-	+
3	Moxidectin	Topical	No	30d, four times	ND	-	+
4	Ivermectin	Oral	Yes	30d, twice	Neg	-	+
5	Ivermectin	Oral	No	30d	Neg	-	+
6	Ivermectin	Oral	No	30d, twice	Neg	-	+
7	Ivermectin	Oral	Yes	30d, twice	Neg	-	+
8	Ivermectin	Oral	No	30d	POS*	-	+
9	Moxidectin	Topical	Yes	6mo	ND	-	-
10	Moxidectin	Topical	No	30d, twice	Neg	-	-
11	Ivermectin	Oral	Yes	30d	Neg	-	-
12	Ivermectin	Oral	Yes	30d, twice	Neg	-	-
13	Ivermectin	Oral	No	30d	ND	-	-
14	Ivermectin,	Oral	Yes	30d, twice	ND	-	-
	Milbemycin	Oral					
15	Ivermectin,	Oral	No	30d, three times	Neg	-	-
	Milbemycin	Oral					

*Identity of microfilariae confirmed as *Dirofilaria immitis* by morphology and by PCR and sequencing.

ND = Not Done.

Evaluation of medical records from each dog revealed SK was performed using monthly low-dose ivermectin (HeartGard Plus®, Merial or IverHeart Plus®, Virbac) in 10 dogs and monthly topical moxidectin/imidicloprid (AdvantageMulti for Dogs®, Bayer Animal Health) in 5 dogs (Table 1). Two of the 10 dogs initially prescribed low-dose ivermectin were switched to a monthly preventive containing milbemycin oxime (Trifexis®, Elanco or Sentinel® Flavor Tabs, Novartis Animal Health) following conversion to negative heartworm antigen status. All 15 dogs were prescribed doxycycline at some point for various periods of time; one dog received 6 months of doxycycline, while all other dogs were prescribed single or multiple, intermittent 30d courses of therapy (Table 1). Based on purchase history, 7 of the 15 dogs had evidence in the medical records supporting compliance with monthly preventive as prescribed.

## Discussion

Together, these data indicate that antigen tests may not be a reliable indicator of heartworm antigen status in *D. immitis*-infected dogs managed with SK; over half the dogs which converted to antigen negative were actually still antigen positive following treatment of serum to disrupt immune complexes. Experimental infection studies have shown that SK prolongs the death of adult worms over 16 months to more than 3 years depending on the age of the worms when the protocol is first initiated [11,12]. During SK, inflammation is induced which is radiographically evident and can exacerbate clinical disease [4,24]. We suspect that the hypergammaglobulinemic state induced by the reaction to dead and dying worms allows formation of antigen-antibody complexes in some canine patients, masking antigen of *D. immitis* and preventing detection on subsequent assays. A simple heat treatment step prior to antigen assay of serum would be expected to destroy any complexes, if present, and allow detection of antigen, the reason this step was routinely included when heartworm antigen tests were first developed [25,26].

A limitation of the present study is that the dogs studied were pet dogs and therefore no necropsy data or worm counts were available. Although over half the dogs testing negative were actually shown to harbor antigen, we may have been detecting residual antigen in some dogs without the concomitant presence of live, adult worms. Similarly, because a second, microtiter well based confirmatory antigen test was not documented for all dogs prior to starting SK, some dogs which apparently converted to true negative may not have been originally infected. Nonetheless, the results of the present study draw attention to the possibility that false negative antigen test results may commonly develop in dogs on SK, a problem which could mislead veterinarians in clinical practice about the infection status of their patients.

One of the dogs in the present study managed with a SK protocol and initially antigen negative had circulating microfilariae (Table 1), while microfilariae were not detected in the other 8 dogs for which whole blood was available; the microfilaria status of the 6 dogs for which only serum was provided is unknown. These data support previous findings suggesting microfilariae continue to be present in some dogs despite monthly administration of macrocyclic lactones [15]. Although not reproduced experimentally, one potentially catastrophic effect of the use of SK is increased selection for resistance to the macrocyclic lactones, the anthelmintic class of all currently available heartworm preventives [15]. This phenomenon was recently investigated by administering repeated doses of ivermectin to a dog infected with macrocyclic lactone-resistant *Dirofilaria immitis,* resulting in an apparent shift in the proportion of microfilariae with resistant markers, a result interpreted to be consistent with the effect of increased selective pressure [27]. Some dispute the importance of the resistance concern by citing research showing that third-stage larvae produced from microfilariae harvested from dogs treated with doxycycline are unable to develop to adult worms [28,29]. While important, those experiments have involved a small number of dogs (n = 1 or 2) challenged with a small number of larvae (n = 5 – 50). Extending the findings to a large population of infected dogs in the field may be imprudent, particularly given the growing awareness of resistance to macrocyclic lactones in *D. immitis* in North America.

Interestingly, the present study also documented that compliance with SK was poor despite a heartworm diagnosis and owner awareness of an active heartworm infection. These data confirm what many veterinarians have long suspected: successfully implementing SK in practice is challenging because it requires a long-term commitment from the pet owner to complete the protocol. Based on review of purchase records alone, over half of the dogs in this study did not complete the SK protocol as prescribed (Table 1). Because the preventive used in SK is intended to protect against future *D. immitis* infection as well as clear existing nematodes^15^, some of the dog in the present study also may have been re-infected due to poor or non-compliance; this reinfection and subsequent development of adult heartworms may have contributed to the presence of blocked antigen in some patients which was revealed only upon heat treatment of serum. Nonetheless, several dogs being managed with SK tested false negative on standard commercial antigen tests, indicating that many negative antigen test results from dogs on SK may not be accurate.

## Conclusions

Managing *D. immitis*-infected dogs with monthly macrocyclic lactones and doxycycline can create false negative results on heartworm antigen tests, misleading veterinarians and pet owners about the efficacy of this approach. The data from the present study indicate that antigen detection as a primary outcome measure is not an accurate means of determining SK success, suggesting previously reported efficacy of SK in pet dogs in clinical practice should be reconsidered. Adulticide therapy with melarsomine dihydrochloride remains the treatment recommended by the Companion Animal Parasite Council, the European Scientific Counsel Companion Animal Parasites, and the American Heartworm Society for dogs infected with adult heartworms.
